# The Preventive Effect of Head Injury by Helmet Type in Motorcycle Crashes: A Rural Korean Single-Center Observational Study

**DOI:** 10.1155/2016/1849134

**Published:** 2016-06-08

**Authors:** Kang-Min Sung, Jennifer Noble, Sang-Chul Kim, Hyeok-Jin Jeon, Jin-Yong Kim, Han-Ho Do, Sang-O Park, Kyeong-Ryong Lee, Kwang-Je Baek

**Affiliations:** ^1^Department of Emergency Medicine, Konkuk University School of Medicine, Konkuk University Chungju Hospital, Chungju 27376, Republic of Korea; ^2^Department of Pediatric Emergency Medicine, Children's Hospital of Michigan, Detroit, MI 48201, USA; ^3^Department of Emergency Medicine, Dongguk University School of Medicine, Dongguk University Ilsan Hospital, Goyang 10326, Republic of Korea; ^4^Department of Emergency Medicine, Konkuk University School of Medicine, Konkuk University Hospital, Seoul 05030, Republic of Korea

## Abstract

*Introduction.* The goal of this study was to determine the preventive effect on head injury by helmet type: full face helmet (FFH), open face helmet (OFH), and half-coverage helmet (HCH).* Methods. *This is a retrospective observational study of motorcycle crash victims between June 2012 and May 2015 in a rural town in Korea. We performed multiple linear regression to predict the effect of each type of helmet compared to unhelmeted status in preventing head injury using dependent variables based on the Abbreviated Injury Scale (AIS) and applied logistic regression modeling to compare the incidence of head injury.* Results.* Of the 738 patients, the number of FFH patients was 33.5%, followed by unhelmeted (27.8%), OFH (17.6%), and HCH (13.0%) patients. The FFH and OFH group had a lower head maximum AIS than unhelmeted group (coefficient: −0.368, 95% CI: −0.559 to −0.177 and coefficient: −0.235, 95% CI: −0.459 to −0.010, resp.) and only FFHs experienced a reduction effect of severe and minor head injury (OR: 0.206, 95% CI: 0.080 to 0.533 and OR: 0.589, 95% CI: 0.377 to 0.920, resp.).* Conclusions.* FFHs and OFHs reduce the risk of head injury, and FFHs have a more preventive effect on head injury in motorcycle crashes.

## 1. Introduction

In high-income countries, motorcycle-related fatalities account for 8–19% of all traffic-related mortalities [1]. There is an increase in fatalities (30–73%) in low- and middle-income countries where motorcycles are used as a major source of transportation [1, 2]. Motorcyclists are more likely to sustain serious injuries in comparison to car drivers since they lack safety devices and have greater environmental exposure. The risk of death among motorcyclists is 30 times greater than that among car drivers, with head injuries the leading cause of death [3, 4].

Fortunately, due to the legal requirement to wear a helmet in many countries, the mortality rate among motorcyclists has decreased along with the rate of head injuries [5–7]. Helmets have been shown to reduce the risk of head injury by 69% and the risk of death by 42% for motorcyclists involved in accidents [8]. Despite helmet laws, however, approximately 41–69% of motorcyclists in low-to-middle-income countries do not wear helmets [9–11]. This may be due to the cost, helmet weight, perceived auditory/visual limitations and/or increased regional temperature, or cultural norms [12]. These factors also may contribute to the use of nonstandard helmets [13, 14].

In South Korea, traffic laws established in 1990 dictate that all motorcyclists must wear a helmet. Traffic laws also prohibit motorcyclists from riding on the highway. Most countries including South Korea, however, have no regulations regarding the type of helmet that must be worn or the appropriate use of helmets. Consequently, various types of helmets are permitted in many countries. Common styles of helmets worn include the full face helmet (FFH), the open face helmet (OFH), and the half coverage helmet (HCH). Previous studies have reported that an FFH is more effective in preventing facial and skull fractures compared with other helmet types [15, 16]. The purpose of this study was to determine whether helmet type had a preventive effect on head injury following a motorcycle crash.

## 2. Methods

This is an observational retrospective cohort study. It was conducted at Konkuk University Chungju Hospital, Korea, which is located in a rural area with a population of 211,000 people and a population density of 214.7 people/km^2^. In 2012, 28,532 patients visited the emergency medical center, including 8,851 (31%) trauma victims and 243 (0.9%) motorcyclists. We collected data about motorcycle crash victims between June 2012 and May 2015. These riders were traveling on both paved and dirt rural roads and none of the incidents occurred on highways. Inclusion criteria were all occupants (driver and passengers) over 15 years of age who were riding a 2-wheel motorcycle involved in a crash, regardless of outcome. Exclusion criteria were patients whose helmet type was unknown and those with incomplete medical records or for whom no injury was recorded.

All patients involved in a motorcycle crash who met inclusion criteria were approached for consent to participate in the study during their hospital course by paramedics trained in the study protocol. Patients or a legally authorized guardian who consented to participate completed a data collection form following the crash. This form included self-reported accident and demographic data: sex, age, rider role, riding speed at the time of the crash, alcohol consumption before the crash, mechanism of the crash, and helmet use and type. Data from police reports and ambulance run sheets if available was collected in an effort to corroborate the veracity of helmet use and determine the type of helmet worn. Electronic medical record and radiologic data were also evaluated to determine abbreviated injury scale (AIS) scoring for each enrolled participant.

Subjects were grouped according to helmet type into FFH, OFH, HCH, and unhelmeted groups. An FFH was defined as a helmet with a chin bar and a face shield that covered the whole head, including the base of the skull. An OFH was defined as a helmet that covered the whole head but did not have a chin bar. An HCH was defined as a helmet that did not have a face shield or chin bar and only covered half of the head.

Type of injury was defined using AIS and the overall severity of injuries was defined using the injury severity score (ISS) from medical records and radiographic images. These values were documented by trauma registry specialists trained in using the AIS scoring system as defined by the American Medical Association Committee on Medical Aspects of Automotive Safety. Trauma registry personnel are blinded in abstracting whole data.

The primary outcome was the preventive effect on head injury of each helmet type compared with unhelmeted status. The secondary outcome was the evaluation of injury severity according to helmet type. We classified dependent variables as no head injury, minor head injury (maximum AIS 1-2), and severe head injury (maximum AIS 3–6) according to AIS.

Statistical testing was performed using SPSS (ver. 23, IBM Corp., Armonk, NY, USA). Continuous variables are expressed as mean ± standard deviation, and categorical variables, such as general patient characteristics, crash information, and outcomes, are expressed as frequency (percentage). Differences between the helmeted group and the unhelmeted group were compared using Student's *t*-test and Pearson's chi-square test. Analysis of variance testing was used to compare age, riding speed, and ISS among groups. Multiple linear regression was performed to predict the effect of each helmet type compared to unhelmeted status in preventing head injury adjusting for potential confounders (sex, age, rider role, riding speed, alcohol consumption, and mechanism of the crash). We excluded 8 riders with unknown values on alcohol consumption, speed or mechanism of crash from the regression model. For comparing the incidence of head injury, we calculated the odds ratios (ORs) using logistic regression model. The criterion for statistical significance was defined as *p* < 0.05.

## 3. Results

Of the 843 patients who were involved in a motorcycle accident and admitted to our hospital during the study period, we excluded 83 (9.8%) subjects who declined to participate in the study, 8 (0.9%) whose helmet type was unknown, and 6 (0.7%) who had inadequate medical data preventing an AIS score from being established. A total of 746 patients were ultimately enrolled (88.5% of those were initially considered candidates).

### 3.1. General Characteristics

Among crash victims, the mean age was 41.3 years, the majority of patients were male drivers who wore helmets with 12.9% of riders reporting alcohol consumption before the crash.  Table 1 compares helmeted and unhelmeted patients by demographics and crash. The proportions of passenger and alcohol consumption in the unhelmeted group were higher than those of the helmeted group (*p* < 0.001). The mechanism of injury was different between the group with helmets and those without helmets (*p* < 0.001).

### 3.2. Prediction of Head Injury according to Helmet Type in Motorcycle Crashes

Using univariate analysis, we evaluated general characteristics according to helmet type and use in motorcycle crashes (Table 2). The mean age of HCH group was the highest in patients who are 53.9 years old. FFH riders had an overall higher speed at the time of the crash than all other groups. The highest number of crashes occurred in patients with FFHs who collided with moving obstacle (motor vehicle or motorcycle).  Table 3 provides the results from a linear regression analysis which demonstrated that FFH and OFH riders had a significantly lower head maximum AIS than unhelmeted riders (mean reduction = −0.368, 95% confidence interval (CI) for mean reduction −0.559 to −0.177, *p* < 0.001 and mean reduction = −0.235, 95% CI for mean reduction −0.459 to −0.010, *p* = 0.040, resp.), while holding age, sex, rider role, riding speed, alcohol consumption, and mechanism of crash constants. Increasing age and riding speed, alcohol consumption, and mechanism of collision with stationary obstacle were associated with more severe head injury as measured by AIS score.

### 3.3. Analysis of the Relationship between Severities of Head Injury and Helmet Type

Using multinomial logistical regression, we evaluated severity of head injury with helmet type. The incidence of head injury was the lowest in the FFH group (minor head injury odds ratio (OR) = 0.589, 95% CI 0.377 to 0.920, and severe head injury OR = 0.206, 95% CI 0.080 to 0.533). The preventive effect of OFH and HCH groups for minor (*p* = 0.188, *p* = 0.513) and severe injury (*p* = 0.055 and *p* = 0.195) were not statistically significant. Older age, higher speed, and alcohol consumption had a higher probability of head injury.

### 3.4. Analysis of the Relationship between ISS and Helmet Type

Using multiple linear regression analysis, FFH and OFH riders had significantly lower ISSs than unhelmeted riders (mean reduction = −2.169, 95% CI −3.251 to −1.088, *p* < 0.001, and mean reduction = −2.008, 95% CI −3.282 to −0.734, *p* = 0.002, resp., Table 5). Age, speed, and alcohol consumption had a significantly positive correlation with ISS.

## 4. Discussion

In this study, we observed that FFH and OFH have a preventive effect on head injury. FFHs are more effective than OFHs and have a preventive effect of severe head injury (OR reduction 79%) and minor head injury (OR reduction 41%). FFHs are known to reduce the incidence of head injury, brain contusion, and craniofacial fractures [15, 17]. In previous research comparing various helmet types, Tsai et al. reported that FFHs were more effective than OFHs in reducing traumatic brain injury (TBI) [18]. In another study that compared three types of helmets after motorcycle accidents, Yu et al. reported that HCHs created a 2.6 times greater risk of head injury compared to FFHs [16]. Our data supports the findings of these studies by showing that, among the helmet groups, FFHs and OFHs were found to be preventive against head injury after motorcycle crashes. In the current study, HCHs did not have a statistically significant preventive effect on head injury.

The study was conducted in a rural area, where regulations regarding standard helmet style or proper use are not strictly enforced [19]. HCHs were used more commonly among older riders. Riders using HCHs were at greater risk for more severe head injuries. This could be because helmets have structural differences which could contribute to the variation in head injury severity after a crash. Additionally, use of borrowed, poorly fitted, or inappropriately secured helmets may result in the helmet being unintentionally removed prior to the crash [16]. For example, in a high-speed crash, a helmet can be knocked off the rider's head if the chin strap is not fully fastened [20]. Interestingly, the prevalence of helmet removal prior to motorcycle accidents is approximately 25% in Thailand, compared to 5% in the USA [21].

The data showed that FFHs and OFHs decrease the ISS, and higher age and speed increase the ISS on the contrary. FFHs' definite preventive effect on head injury could affect the decrease of overall body injury. In the case of OFHs, some amount of preventive effect of head could affect the decrease in the ISS (*p* = 0.040, Table 3, and *p* = 0.055 at severe head injury, Table 4). Other studies reported that there was no difference in the whole body injury between FFH riders and OFH riders [15, 17]. In addition, older age and high motor cycle speed were strong predictors of body injury as well as head injury [22, 23].

Although low- and middle-income countries often have a high prevalence of motorcycle use, the rate of helmet usage in these countries is low (31–59%) [9–11]. In Southeast Asia and Africa, helmets are considered a burden, and the hot weather is an obstacle to mandated helmet use [24, 25]. Therefore, motorcycle helmets should be designed to reflect the climate conditions of each country while also serving the ultimate function of protecting against head injury. Additionally, safety devices such as protective jackets, pants, or gloves can decrease injury. Preliminary studies of motorcycle airbags also suggest a reduction in rider injuries [26–28].

This study had several limitations. First, the study was conducted in a single local rural emergency center creating a selection bias. Patients who could walk home from the incident or who had minor injuries were excluded because they did not come to the ED following the injury. In addition, we did not collect police report data on driver speed, on whether the helmet was worn appropriately, or on whether the patient was impaired from alcohol. We relied on patient recall and veracity in documenting the vehicle incident and did not corroborate this information with police records. Finally, we excluded 8 riders from the regression analysis from the overall 746 evaluated due to unknown values on their alcohol consumption, riding speed, and mechanism of crash. In this group including 5 unhelmeted riders and 2 HCH riders, all cases resulted in death.

## 5. Conclusions

The findings from this study suggest that FFHs and OFHs reduce the risk of head injury, and FFHs have a protective effect against minor and severe head injury. Motorcyclists should wear appropriately fitting FFH or OFH. Legislative efforts also should be taken to eliminate use of novelty helmets.

## Figures and Tables

**Table 1:** Comparison of general characteristics between helmeted group and unhelmeted group in motorcycle crashes.

Category	Total, *n* = 746	Helmeted, *n* = 509	Unhelmeted, *n* = 237	*p* value
	*n* (%)	*n* (%)	*n* (%)	
Sex				
Male	650 (87.1)	455 (89.4)	195 (82.3)	0.007
Female	96 (12.9)	54 (10.6)	42 (17.7)	
Age, mean ± SD (years)	41.3 ± 23.6	42.9 ± 23.1	37.8 ± 24.2	0.006
Rider role				
Driver	681 (91.3)	496 (97.4)	185 (78.1)	<0.001
Passenger	65 (8.7)	13 (2.6)	52 (21.9)	
Riding speed, mean ± SD (Km/h)	39.4 ± 19.9	39.8 ± 20.7	38.4 ± 17.9	0.371
Alcohol consumption before the crash				
Yes	92 (12.9)	45 (8.8)	47 (19.8)	<0.001
No	648 (86.9)	462 (90.8)	186 (78.5)	
Unknown	6 (0.8)	2 (0.4)	4 (1.7)	
Mechanism of crash				
MC collision with stationary obstacle	60 (8.0)	40 (7.9)	20 (8.4)	<0.001
No object	209 (28.0)	118 (23.4)	90 (38.0)	
MC collision with moving obstacle	305 (40.9)	223 (43.8)	82 (34.6)	
MV collision with MC	167 (22.4)	126 (24.8)	41 (17.3)	
Unknown	5 (0.7)	1 (0.2)	4 (1.7)	

SD: standard deviation; MC: motorcycle; and MV: motor vehicle.

Categorical variables between helmeted group and unhelmeted group were compared using the chi-square test, and continuous variables were compared using Student's *t*-test.

**Table 2:** Univariate analysis of general characteristics according to helmet type and use in motorcycle crashes (*N* = 738).

Category	Full face *n* = 261, *n* (%)	Open face *n* = 135, *n* (%)	Half coverage *n* = 110, *n* (%)	Unhelmeted *n* = 232, *n* (%)	*p* value
Sex					
Male	247 (94.6)	113 (83.7)	92 (83.6)	191 (82.3)	<0.001
Female	14 (5.4)	22 (16.3)	18 (16.4)	41 (17.7)	
Age, mean ± SD (years)	34.9 ± 20.7	49.0 ± 23.1	53.9 ± 21.7	37.4 ± 23.9	<0.001
Rider role					
Driver	254 (97.3)	130 (96.3)	109 (99.1)	181 (78.0)	<0.001
Passenger	7 (2.7)	5 (3.7)	1 (0.9)	51 (22.0)	
Riding speed, mean ± SD (Km/h)	44.0 ± 22.9	34.6 ± 15.0	37.1 ± 18.9	38.7 ± 17.3	<0.001
Alcohol consumption before the crash					
Yes	18 (6.9)	14 (10.4)	13 (11.8)	47 (20.3)	<0.001
No	243 (93.1)	121 (89.6)	97 (88.2)	185 (79.7)	
Mechanism of crash					
MC collision with stationary obstacle	16 (6.1)	11 (8.1)	13 (11.8)	19 (8.2)	0.003
No object	62 (23.8)	34 (25.2)	22 (20.0)	90 (38.8)	
MC collision with moving obstacle	123 (47.1)	58 (43.0)	41 (37.3)	82 (35.3)	
MV collision with MC	60 (23.0)	32 (23.7)	34 (30.9)	41 (17.7)	
ISS, mean (95% CI)	4.34 (3.68–4.99)	5.19 (4.33–6.04)	6.94 (5.63–8.25)	6.03 (4.99–7.07)	0.002

MC: motorcycle; MV: motor vehicle.

Categorical variables among groups were compared using the chi-square test, and continuous variables were compared using the analysis of variance.

**Table 3:** Multiple linear regression analysis of head abbreviated injury scale.

	*B*	95% confidence intervals		*p* value
		Lower	Upper	
Helmet type				
Unhelmeted	Reference			
Full face helmet	−0.368	−0.559	−0.177	<0.001
Open face helmet	−0.235	−0.459	−0.010	0.040
Half coverage helmet	−0.204	−0.449	0.040	0.101
Sex				
Male	Reference			
Female	0.185	−0.106	0.475	0.213
Age (years)	0.017	0.013	0.020	<0.001
Rider role				
Passenger	Reference			
Driver	0.185	−0.106	0.475	0.213
Riding speed (Km/h)	0.014	0.010	0.018	<0.001
Alcohol consumption				
No	Reference			
Yes	0.382	0.147	0.617	0.001
Mechanism of crash				
MV collision with MC	Reference			
MC collision with stationary obstacle	0.322	0.016	0.628	0.039
No object	0.038	−0.176	0.253	0.725
MC collision with moving obstacle	0.108	−0.084	0.300	0.272

MV: motor vehicle; MC: motorcycle.

A multiple linear regression analysis was performed based on helmet type, sex, age, occupant role, riding speed, alcohol, and mechanism of crash.

**Table 4:** Multinomial logistic regression analysis of head injury severity.

	Minor head injury	*p* value	Severe head injury	*p* value
	OR (95% CI)		OR (95% CI)	
Helmet type				
Unhelmeted	Reference		Reference	
Full face helmet	0.589 (0.377–0.920)	0.020	0.206 (0.080–0.533)	0.001
Open face helmet	0.705 (0.420–1.185)	0.188	0.404 (0.160–1.021)	0.055
Half coverage helmet	0.831 (0.477–1.447)	0.513	0.569 (0.243–1.335)	0.195
Sex				
Male	Reference		Reference	
Female	1.266 (0.747–2.148)	0.381	1.901 (0.741–4.878)	0.182
Age (years)	1.019 (1.011–1.027)	<0.001	1.069 (1.050–1.088)	<0.001
Rider role				
Passenger	Reference		Reference	
Driver	1.321 (0.671–2.603)	0.420	1.891 (0.509–7.023)	0.341
Riding speed (Km/h)	1.014 (1.005–1.024)	0.003	1.053 (1.036–1.070)	<0.001
Alcohol consumption				
No	Reference		Reference	
Yes	2.298 (1.369–3.855)	0.002	2.828 (1.184–6.757)	0.019
Mechanism of crash				
MV collision with MC	Reference		Reference	
MC collision with stationary obstacle	1.540 (0.758–3.132)	0.233	2.811 (0.922–8.568)	0.069
No object	1.162 (0.712–1.962)	0.517	0.742 (0.281–1.963)	0.548
MC collision with moving obstacle	1.318 (0.835–2.081)	0.236	1.153 (0.486–2.736)	0.747

OR: odds ratio; MC: motorcycle; MV: motor vehicle; and AIS: abbreviated injury scale.

We divided subjects into 3 groups according to AIS as follows: no head injury (head maximum AIS = 0), minor head injury (head maximum AIS = 1, 2), and severe head injury (3 ≤ head maximum AIS ≤ 6). A multinomial logistic regression was performed adjusting for sex, age, rider role, riding speed, alcohol consumption, and mechanism of crashes. Reference injury category was no head injury.

**Table 5:** Multiple linear regression analysis of injury severity score.

	*B*	95% confidence intervals		*p* value
		Lower	Upper	
Helmet type				
Unhelmeted	Reference			
Full face helmet	−2.169	−3.251	−1.088	<0.001
Open face helmet	−2.008	−3.282	−0.734	0.002
Half coverage helmet	−1.204	−2.590	0.182	0.089
Sex				
Male	Reference			
Female	−0.351	−1.685	0.982	0.605
Age	0.118	0.099	0.137	<0.001
Rider role				
Passenger	Reference			
Driver	1.261	−0.389	2.911	0.134
Riding speed at the crash (Km/h)	0.104	0.082	0.126	<0.001
Alcohol consumption				
No	Reference			
Yes	1.343	0.011	2.676	0.048
Mechanism of crash				
MV collision with MC	Reference			
MC collision with stationary obstacle	1.129	−0.609	2.866	0.203
No object	−0.692	−1.907	0.523	0.264
MC collision with moving obstacle	0.301	−0.789	1.390	0.589

MV: motor vehicle; MC: motorcycle.

A multiple linear regression analysis was performed based on helmet type, sex, age, riding speed, alcohol consumption, occupant role, and mechanism of crash.

## References

[1] International Traffic Safety Data and Analysis Group http://www.internationaltransportforum.org.

[2] Bachani A. M., Tran N. T., Sann S. (2012). Helmet use among motorcyclists in cambodia: a survey of use, knowledge, attitudes, and practices. *Traffic Injury Prevention*.

[3] Lin M.-R., Kraus J. F. (2009). A review of risk factors and patterns of motorcycle injuries. *Accident Analysis and Prevention*.

[4] Sosin D. M., Sacks J. J., Holmgreen P. (1990). Head injury-associated deaths from motorcycle crashes: relationship to helmet-use laws. *Journal of the American Medical Association*.

[5] Sosin D. M., Sacks J. J. (1992). Motorcycle helmet-use laws and head injury prevention. *Journal of the American Medical Association*.

[6] Servadei F., Begliomini C., Gardini E., Giustini M., Taggi F., Kraus J. (2003). Effect of Italy's motorcycle helmet law on traumatic brain injuries. *Injury Prevention*.

[7] Chiu W.-T., Kuo C.-Y., Hung C.-C., Chen M. (2000). The effect of the Taiwan motorcycle helmet use law on head injuries. *American Journal of Public Health*.

[8] Liu B. C., Ivers R., Norton R., Boufous S., Blows S., Lo S. K. (2008). Helmets for preventing injury in motorcycle riders. *Cochrane Database of Systematic Reviews*.

[9] Akaateba M. A., Amoh-Gyimah R., Yakubu I. (2014). A cross-sectional observational study of helmet use among motorcyclists in Wa, Ghana. *Accident Analysis & Prevention*.

[10] Khan I., Khan A., Aziz F., Islam M., Shafqat S. (2008). Factors associated with helmet use among motorcycle users in Karachi, Pakistan. *Academic Emergency Medicine*.

[11] Sreedharan J., Muttappillymyalil J., Divakaran B., Haran J. C. (2010). Determinants of safety helmet use among motorcyclists in Kerala, India. *Journal of Injury & Violence Research*.

[12] Tsui C. K., Rice T. M., Pande S. (2014). Predictors of nonstandard helmet use among San Francisco Bay-area motorcyclists. *Traffic Injury Prevention*.

[13] Moghisi A., Mohammadi R., Svanström L. (2014). Impact of safe community program on motorcyclists' safety with focus on helmet usage in 14 cities of IR Iran. *International Journal of Injury Control and Safety Promotion*.

[14] Ackaah W., Afukaar F., Agyemang W. (2013). The use of non-standard motorcycle helmets in low- and middle-income countries: a multicentre study: road traffic injuries research network multicenter study collaborators. *Injury Prevention*.

[15] Brewer B. L., Diehl A. H., Johnson L. S. (2013). Choice of motorcycle helmet makes a difference: a prospective observational study. *Journal of Trauma and Acute Care Surgery*.

[16] Yu W.-Y., Chen C.-Y., Chiu W.-T., Lin M.-R. (2011). Effectiveness of different types of motorcycle helmets and effects of their improper use on head injuries. *International Journal of Epidemiology*.

[17] Hitosugi M., Shigeta A., Takatsu A., Yokoyama T., Tokudome S. (2004). Analysis of fatal injuries to motorcyclists by helmet type. *The American Journal of Forensic Medicine and Pathology*.

[18] Tsai Y.-J., Wang J.-D., Huang W.-F. (1995). Case-control study of the effectiveness of different types of helmets for the prevention of head injuries among motorcycle riders in Taipei, Taiwan. *American Journal of Epidemiology*.

[19] Xuequn Y., Ke L., Ivers R., Du W., Senserrick T. (2011). Prevalence rates of helmet use among motorcycle riders in a developed region in China. *Accident Analysis and Prevention*.

[20] Richter M., Otte D., Lehmann U. (2001). Head injury mechanisms in helmet-protected motorcyclists: prospective multicenter study. *The Journal of Trauma*.

[21] Ouellet J. V., Kasantikul V. (2006). Motorcycle helmet effect on a per-crash basis in Thailand and the United States. *Traffic Injury Prevention*.

[22] Erhardt T., Rice T., Troszak L., Zhu M. (2016). Motorcycle helmet type and the risk of head injury and neck injury during motorcycle collisions in California. *Accident Analysis and Prevention*.

[23] Lin M.-R., Chang S.-H., Huang W., Hwang H.-F., Pai L. (2003). Factors associated with severity of motorcycle injuries among young adult riders. *Annals of Emergency Medicine*.

[24] de Rome L., Ivers R., Fitzharris M. (2011). Motorcycle protective clothing: protection from injury or just the weather?. *Accident Analysis & Prevention*.

[25] De Rome L., Ivers R., Fitzharris M., Haworth N., Heritier S., Richardson D. (2012). Effectiveness of motorcycle protective clothing: riders' health outcomes in the six months following a crash. *Injury*.

[26] Aikyo Y., Kobayashi Y., Akashi T., Ishiwatari M. (2015). Feasibility study of airbag concept applicable to motorcycles without sufficient reaction structure. *Traffic Injury Prevention*.

[27] Solagberu B. A., Ofoegbu C. K. P., Nasir A. A., Ogundipe O. K., Adekanye A. O., Abdur-Rahman L. O. (2006). Motorcycle injuries in a developing country and the vulnerability of riders, passengers, and pedestrians. *Injury Prevention*.

[28] Nwadiaro H. C., Ekwe K. K., Akpayak I. C., Shitta H. (2011). Motorcycle injuries in north-central Nigeria. *Nigerian Journal of Clinical Practice*.

